# Supplementary material for: Multi-omics prediction of immune-related adverse events during checkpoint immunotherapy
Source: Nat Commun. 2020 Oct 2;11:4946. doi: 10.1038/s41467-020-18742-9 (PMC7532211; doi:10.1038/s41467-020-18742-9)
Supplement: Supplementary file 1 — Supplementary Information [file 41467_2020_18742_MOESM1_ESM.pdf]

Multi-omics prediction of immune-related adverse events during checkpoint immunotherapy

Ying Jing, Jin Liu, Youqiong Ye, Lei Pan, Hui Deng, Yushu Wang, Yang Yang, Lixia Diao, Steven H. Lin, Gordon B. Mills, Guanglei Zhuang*, Xinying Xue*, Leng Han*

Supplementary Information

Supplementary Figure 1

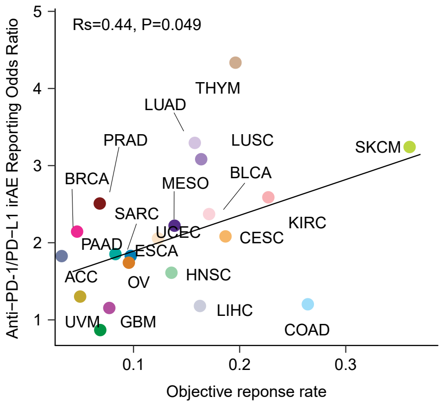

Supplementary Figure 1. Spearman correlation between irAE ROR and objective response rate across 21 cancer types. irAE: immune related adverse events; ROR: reporting odds ratio. LUAD, lung adenocarcinoma; SKCM, skin cutaneous melanoma; LUSC, lung squamous cell carcinoma; KIRC, kidney renal clear cell carcinoma; PRAD, prostate adenocarcinoma; BLCA, bladder urothelial carcinoma; MESO, mesothelioma; BRCA, breast invasive carcinoma; CESC, cervical squamous cell carcinoma and endocervical adenocarcinoma; UCEC, uterine corpus endometrial carcinoma; SARC, sarcoma; ESCA, esophageal carcinoma; PAAD, pancreatic adenocarcinoma; OV, ovarian serous cystadenocarcinoma; HNSC, head and neck squamous cell carcinoma; STAD, stomach adenocarcinoma; THCA, thyroid carcinoma; CHOL, cholangiocarcinoma; ACC, adrenocortical carcinoma; READ, rectum adenocarcinoma; COAD, colon adenocarcinoma; LIHC, liver hepatocellular carcinoma; LGG, brain lower grade glioma; GBM, glioblastoma multiforme; UVM, uveal melanoma; UCS, uterine carcinosarcoma;

Supplementary Figure 2

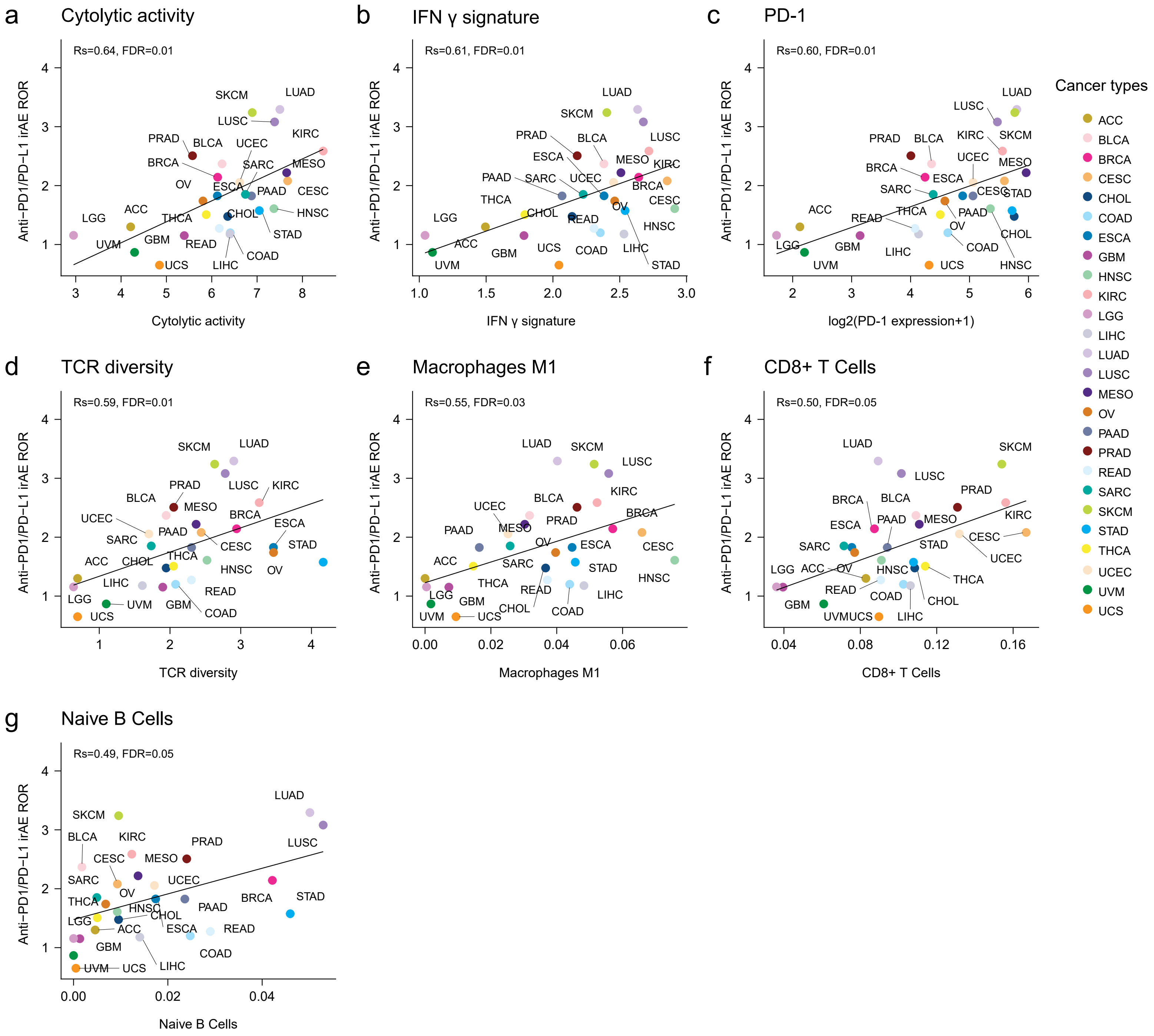

Supplementary Figure 2. For anti-PD-1/PD-L1 therapy across cancer types, the Spearman correlation between irAE ROR and (**a**) cytolytic activity, (**b**) IFN γ signature, (**c**) PD-1 expression, (**d**) TCR diversity,(**e**) estimated Macrophages M1 abundance, (**f**) estimated CD8+ T cell abundance, and (**g**) estimated Naive B Cell abundance. The dot color represents the cancer type.

Supplementary Figure 3

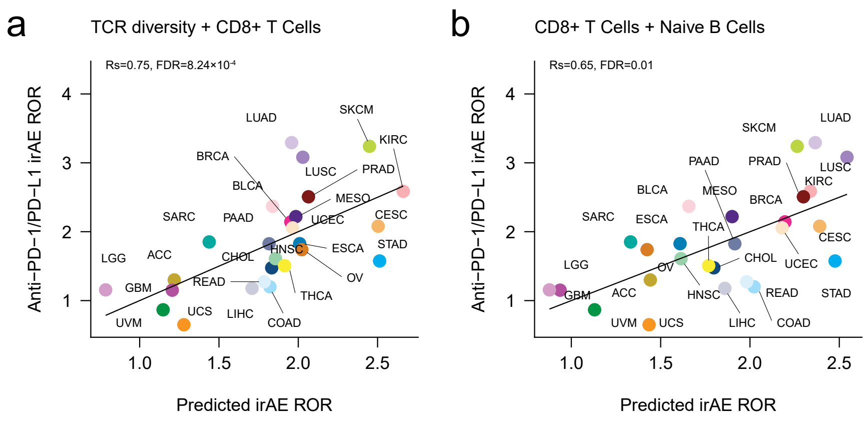

Supplementary Figure 3. Combined effects of bivariate model for all combinations of seven significantly correlated factors. Only models with significant log-likelihood ratio test p-values are plotted.

Supplementary Figure 4

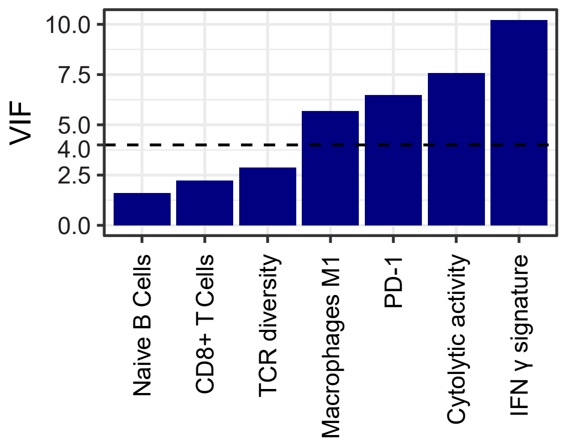

Supplementary Figure 4. Variance inflation factor (VIF) value of seven significant factors associated with irAE ROR.

Supplementary Figure 5

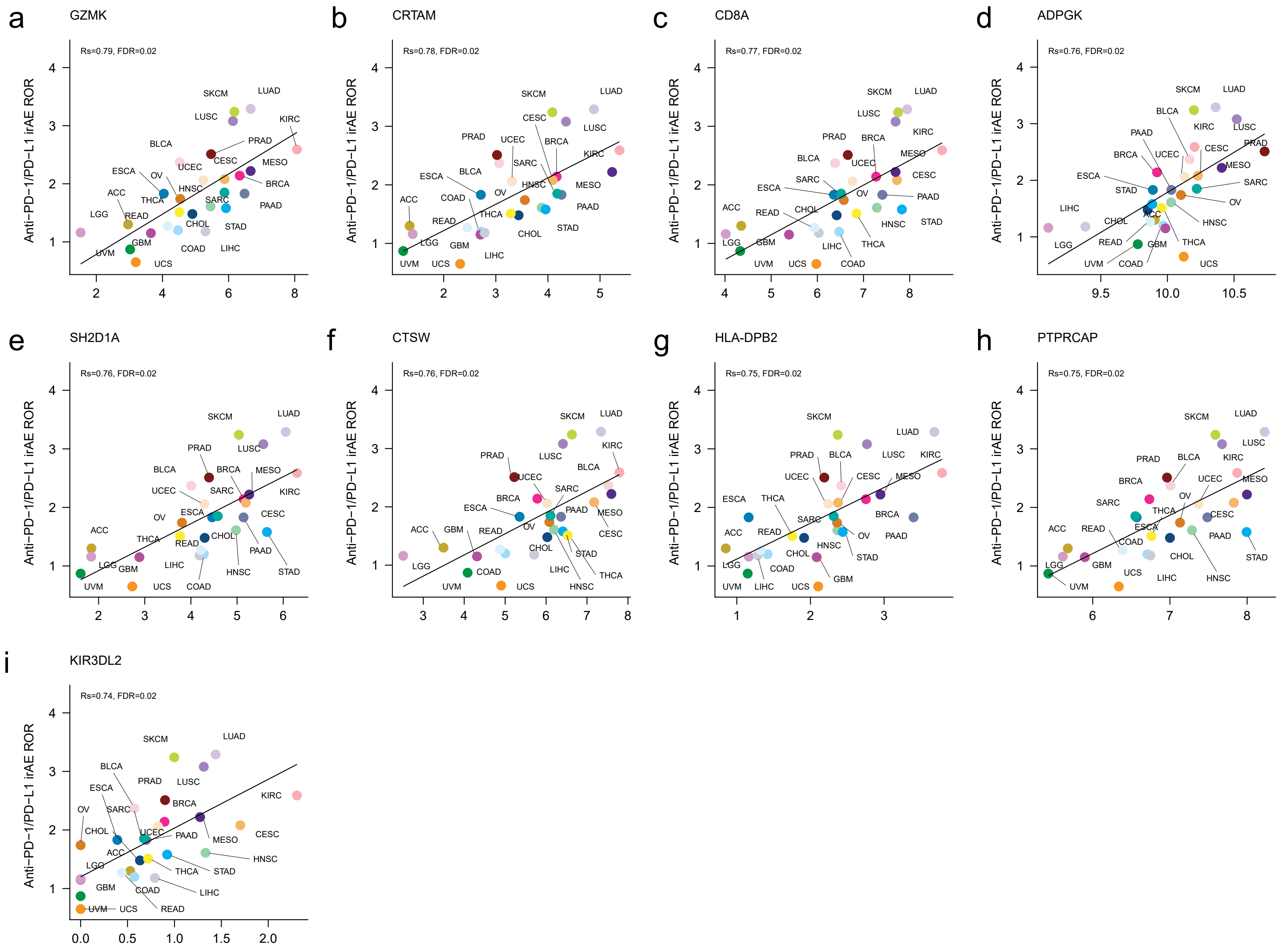

Supplementary Figure 5. Spearman correlation between irAE ROR and top 10 significantly correlated genes (except LCP1). X-axis is the log2 transformed gene expression.

Supplementary Figure 6

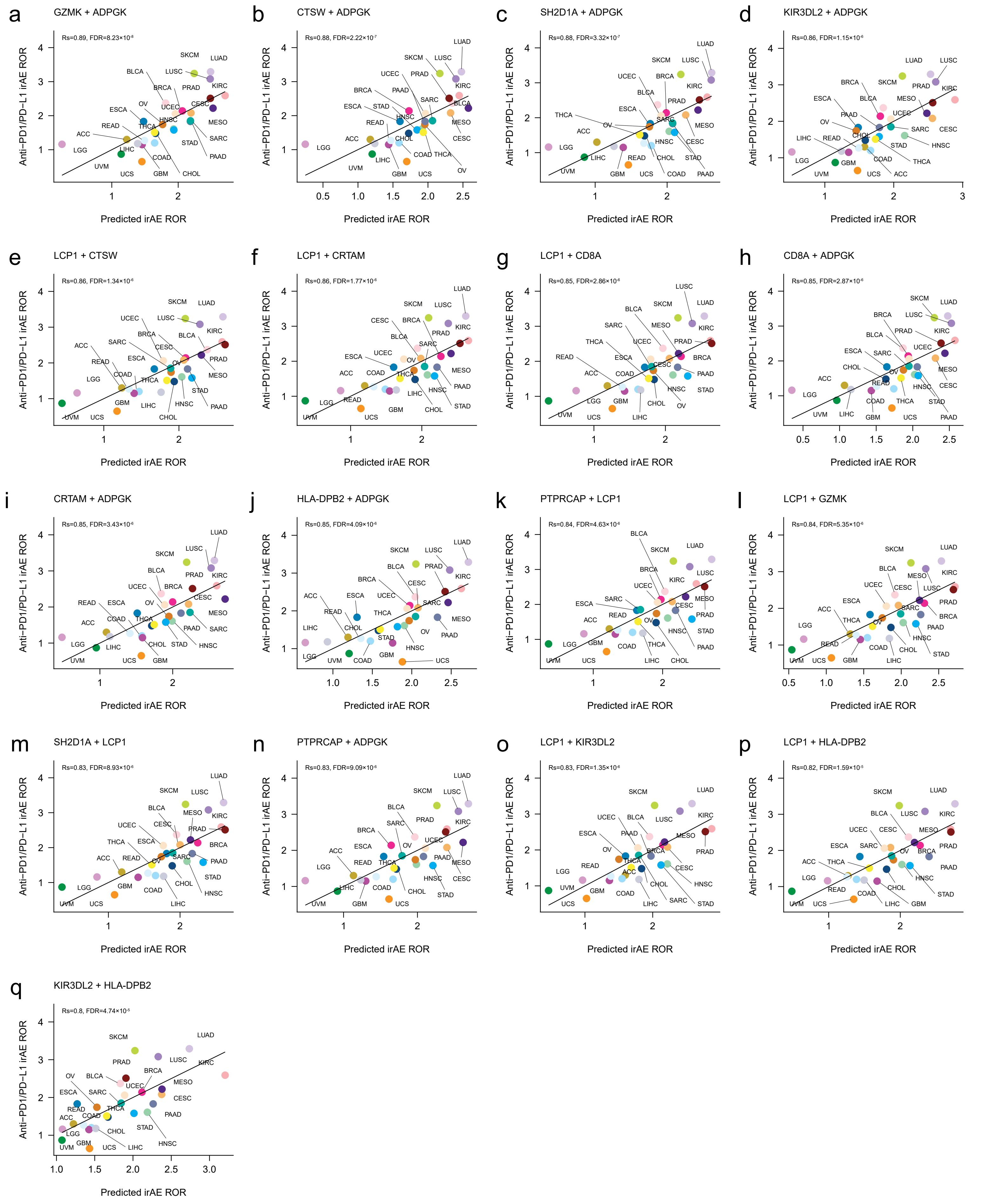

Supplementary Figure 6. Combined effects of the bivariate model for all combinations of the top 10 significantly correlated genes. Only models with significant log-likelihood ratio test p-values are plotted.

Supplementary Figure 7

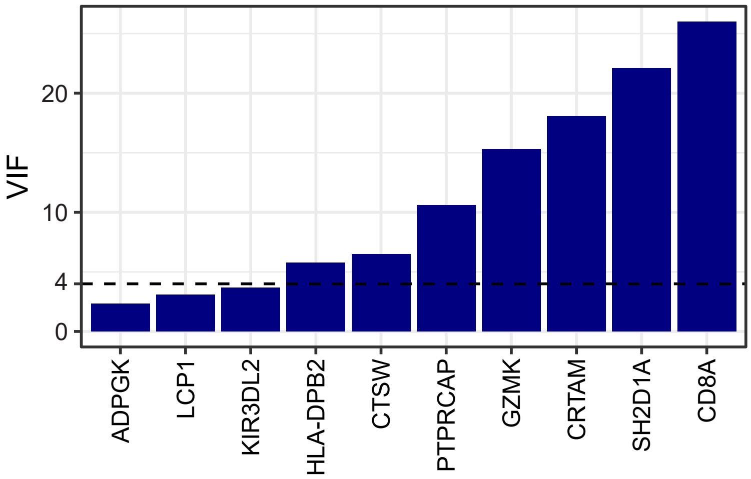

Supplementary Figure 7. Variance inflation factor (VIF) value of top 10 significantly correlated genes.

Supplementary Figure 8

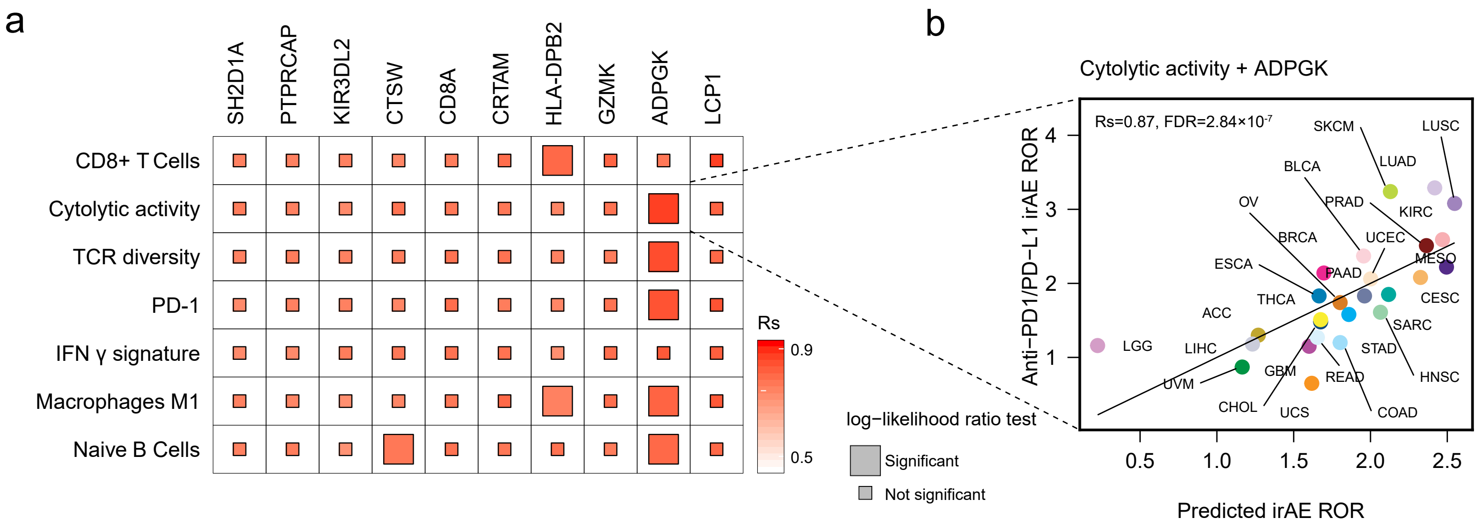

Supplementary Figure 8. Comparison of performance of bivariate models in predicting irAE for combinations of significant top 7 factors and top 10 genes for irAE prediction. **a.** Spearman correlation (Rs) was calculated between the predicted and observed irAE ROR. The shade of the square indicates the Rs, and the size indicates the significance of the log-likelihood ratio test. **b.** Combined effect of CD8+ T cells and LCP1 bivariate model (Spearman correlation, *Rs*=0.87, *FDR*=2.84×10^{-7}).

Supplementary Figure 9

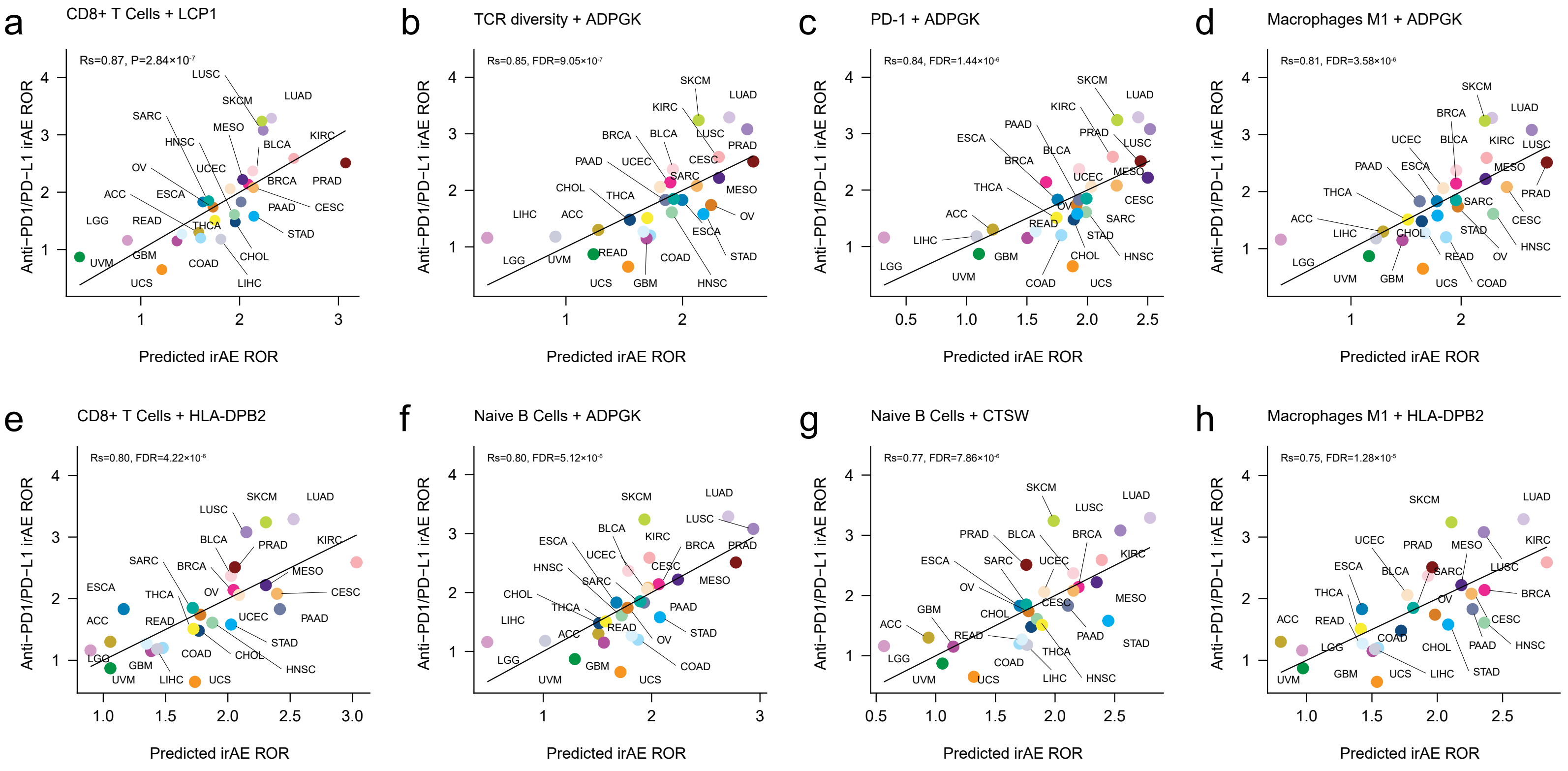

Supplementary Figure 9. Combined effects of the bivariate model for all combinations of top 7 factors and top 10 significantly correlated genes. Only models with significant log-likelihood ratio test p-values are plotted.

Supplementary Figure 10

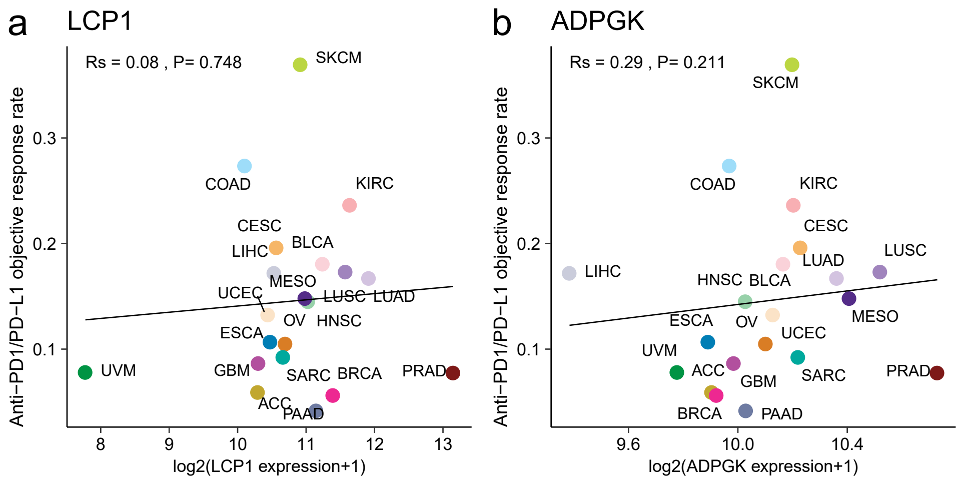

Supplementary Figure 10. Spearman correlation between anti-PD-1/PD-L1 objective response rate and LCP1 and ADPGK expression.

Supplementary Figure 11

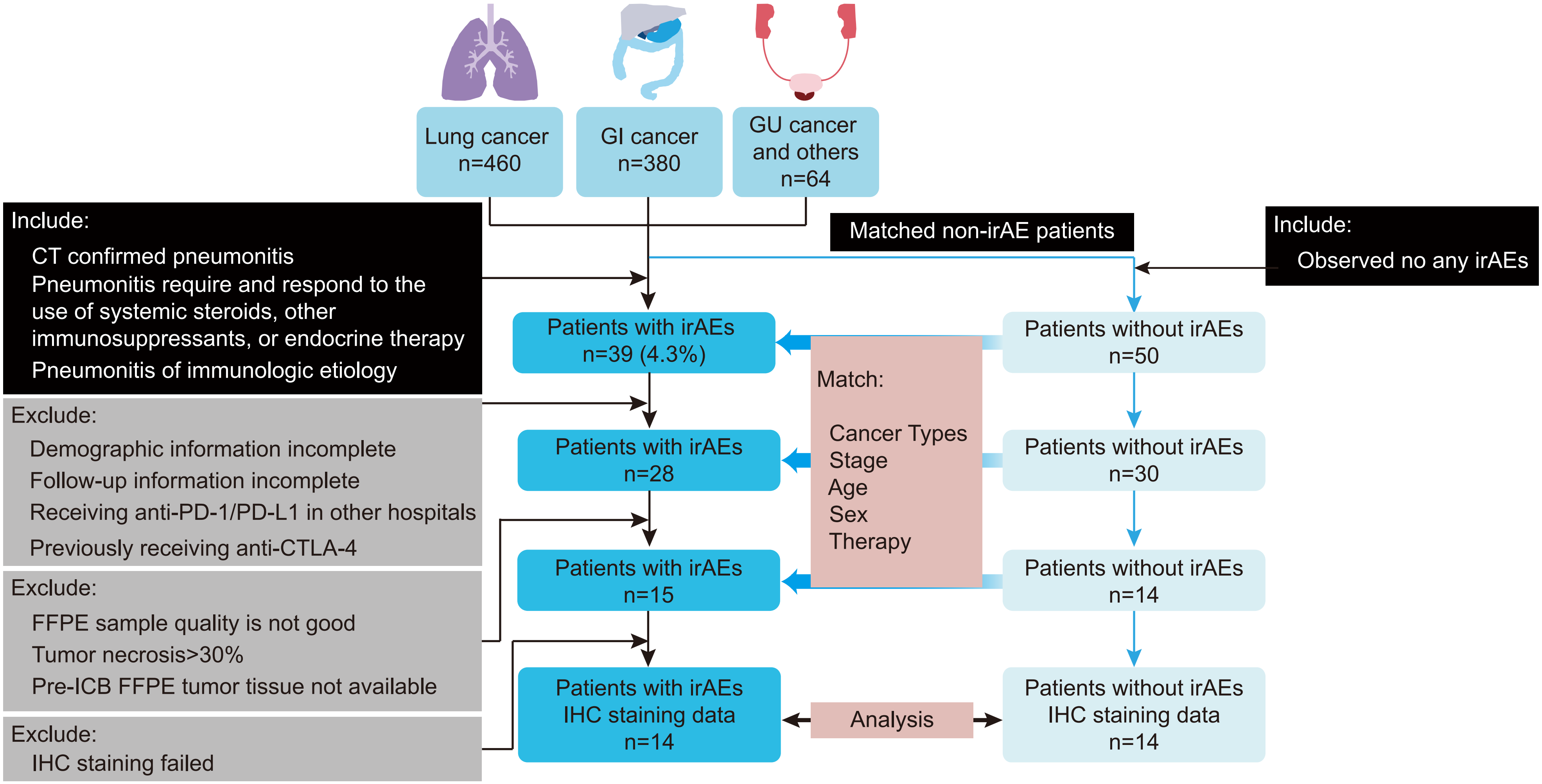

Supplementary Figure 11. Diagram for the sample collection of validation cohort.
Gastrointestinal (GI) cancer include gastric, liver, colorectal and gallbladder cancer; Genitourinary (GU) cancer include kidney, bladder, prostate cancer.

Supplementary Figure 12

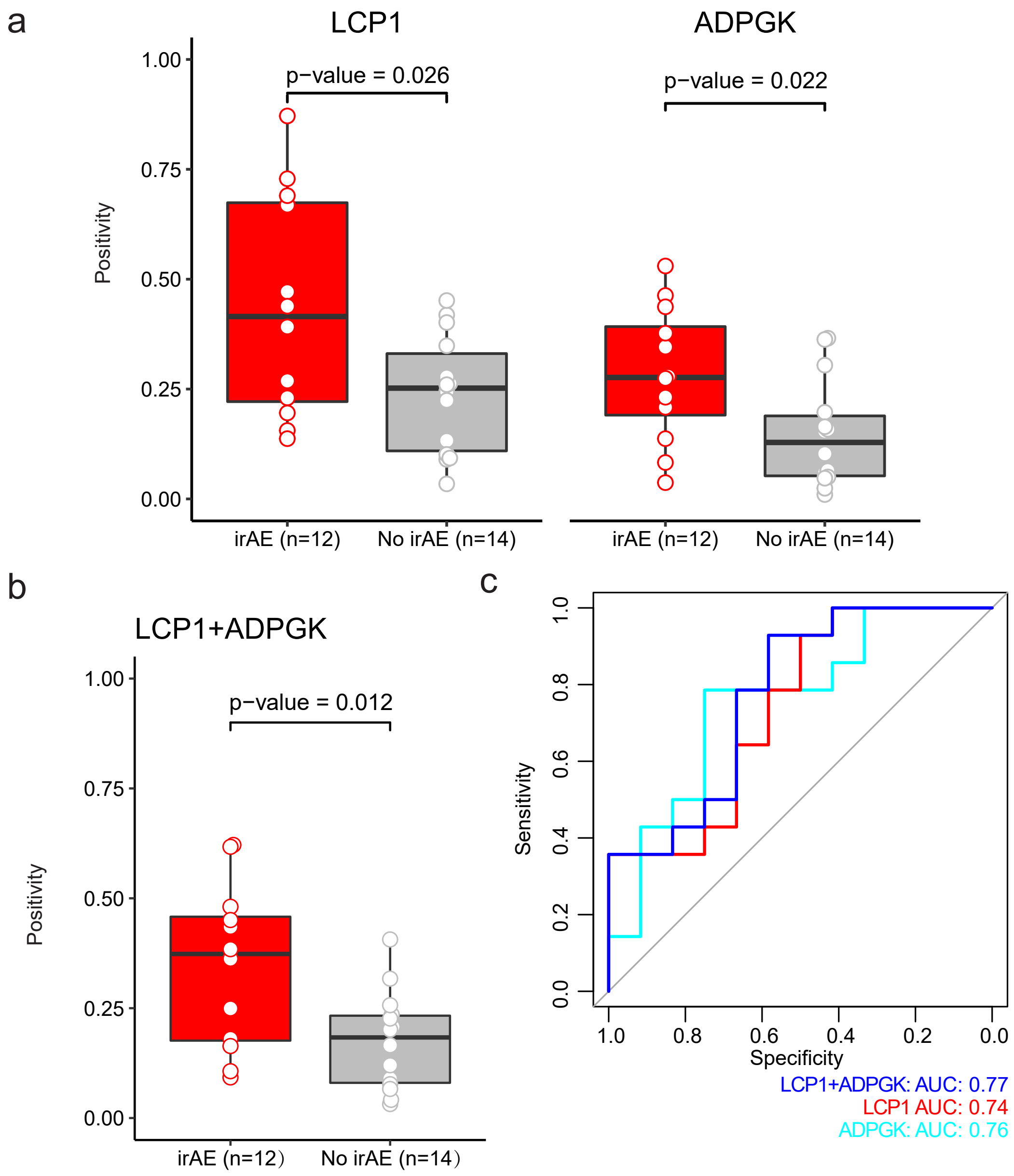

Supplementary Figure 12. Validation of the predictive power of LCP1 and ADPGK in lung cancer patients cohort. a. Quantification of LCP1 and ADPGK IHC staining signals in lung cancer patients. Comparison between patients with irAE or without irAE is conducted by unpaired two-sided student's t-test. b. Geometric mean of LCP1 and ADPGK staining signals. Comparison between patients with irAE and without irAE is conducted by unpaired two-sided student's t-test. c. ROC curve of the LCP1, ADPGK, LCP1+ADPGK in the lung cancer patient cohort (n=26). The boxes indicate the median ±1 quartile, with whiskers extending from the hinge to the smallest or largest value within 1.5 interquartile range from the box boundaries. ROC: receiver operating characteristic; AUC: Area under the receiver operating characteristic curve.

Supplementary Figure 13

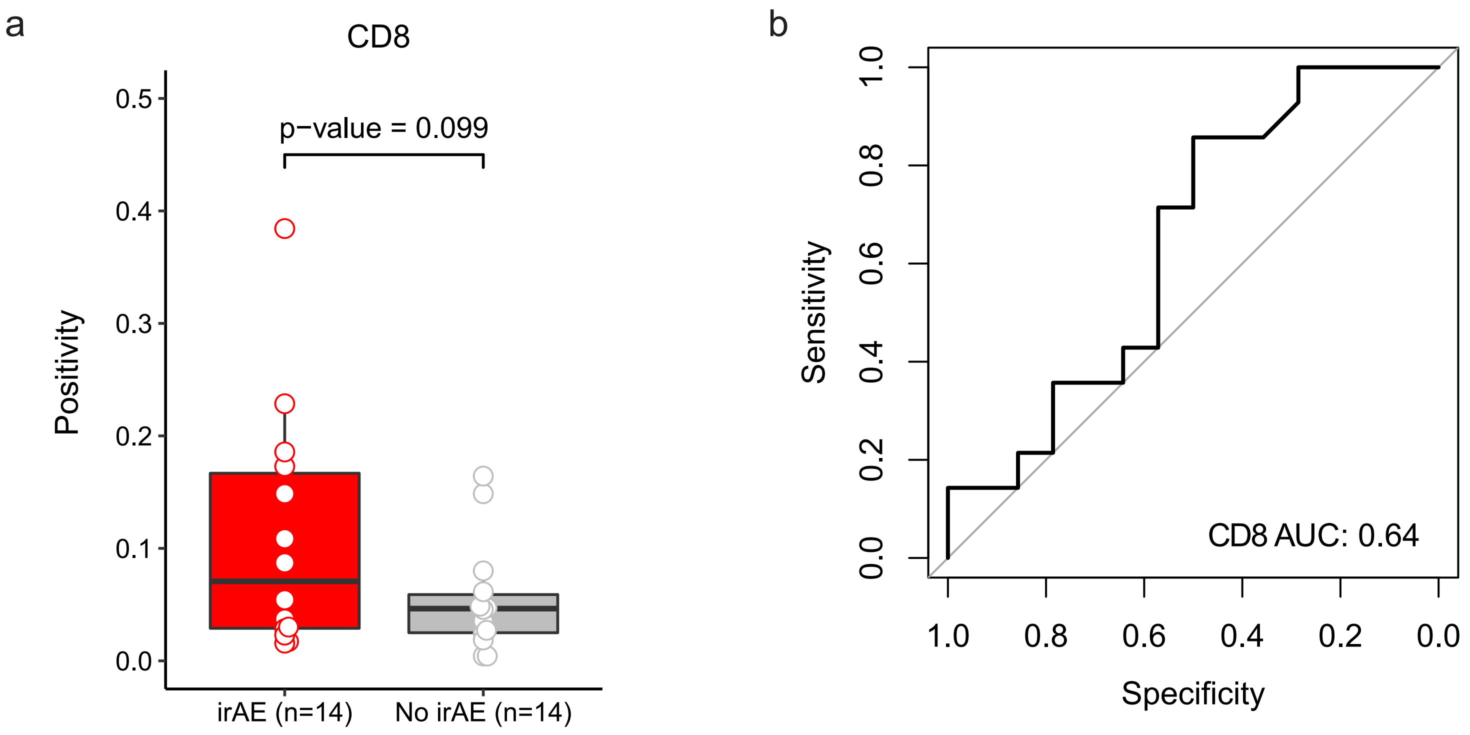

Supplementary Figure 13. Estimation of the predictive power of CD8 in a patient cohort. **a.** Quantification of CD8 IHC staining signals. Comparison between patients with irAE or without irAE is conducted by unpaired two-sided student's t-test. **b.** ROC curve of the CD8 in the validation cohort (n=28). The boxes indicate the median ±1 quartile, with whiskers extending from the hinge to the smallest or largest value within 1.5 interquartile range from the box boundaries. ROC: receiver operating characteristic; AUC: Area under the receiver operating characteristic curve.

Supplementary Table 1. IrAE reporting odds ratio (ROR) and information across 26 cancer types

Cancer types Abbreviation	Full name of Cancer types	FAERS				TCGA sample size			
		irAE ROR	sample size	95%CI		mRNA	Somatic muataion	Protein	miRNA
LUAD	Lung adenocarcinoma	3.29	2001	2.97	3.65	515	533	362	448
SKCM	Skin Cutaneous Melanoma	3.24	6400	3.06	3.43	470	290	353	378
LUSC	Lung squamous cell carcinoma	3.08	919	2.63	3.6	502	177	325	336
KIRC	Kidney renal clear cell carcinoma	2.59	2848	2.36	2.84	533	441	445	240
PRAD	Prostate adenocarcinoma	2.51	278	1.83	3.39	497	425	351	491
BLCA	Bladder Urothelial Carcinoma	2.37	221	1.64	3.34	408	394	344	409
MESO	Mesothelioma	2.22	290	1.61	3.01	87	82	61	87
BRCA	Breast invasive carcinoma	2.14	890	1.79	2.55	1097	975	874	749
CESC	Cervical squamous cell carcinoma and endocervical adenocarcinoma	2.08	144	1.28	3.24	305	194	171	306
UCEC	Uterine Corpus Endometrial Carcinoma	2.06	200	1.37	3.00	177	248	404	351
SARC	Sarcoma	1.85	225	1.25	2.68	259	247	221	256
ESCA	Esophageal carcinoma	1.83	328	1.32	2.48	184	185	126	182
PAAD	Pancreatic adenocarcinoma	1.83	355	1.34	2.45	178	146	105	177
OV	Ovarian serous cystadenocarcinoma	1.74	649	1.38	2.17	305	465	411	310
HNSC	Head and Neck squamous cell carcinoma	1.61	530	1.23	2.07	520	510	346	374
STAD	Stomach adenocarcinoma	1.58	783	1.27	1.94	415	289	392	387
THCA	Thyroid carcinoma	1.51	79	0.69	2.94	505	401	372	502
CHOL	Cholangiocarcinoma	1.48	185	0.91	2.29	36	35	30	36
ACC	Adrenocortical carcinoma	1.30	9	0.03	9.70	79	62	46	79
READ	Rectum adenocarcinoma	1.27	55	0.45	2.98	94	122	130	89
COAD	Colon adenocarcinoma	1.20	232	0.75	1.84	286	367	357	251
LIHC	Liver hepatocellular carcinoma	1.18	688	0.91	1.51	371	198	184	369
LGG	Brain Lower Grade Glioma	1.16	10	0.03	8.34	516	513	427	510
GBM	Glioblastoma multiforme	1.15	331	0.78	1.65	161	283	205	0
UVM	Uveal Melanoma	0.87	39	0.17	2.74	80	80	12	80
UCS	Uterine Carcinosarcoma	0.65	17	0.02	4.18	57	57	48	56

Supplementary Table 2.Spearman correlation between 36 irAE/immune related factors and irAE ROR

irAE/immune therapy response related factors	Category	Rs	P	FDR
Cytolytic activity	Immune therapy response related factor	0.64	5.60E-04	0.01
IFN γ signature	Immune therapy response related factor	0.61	1.14E-03	0.01
PD-1	Immune therapy response related factor	0.60	1.57E-03	0.01
TCR diversity	irAE related factor/Immune therapy response related factor	0.59	1.45E-03	0.01
Macrophages M1	Immune therapy response related factor	0.55	3.92E-03	0.03
CD8+ T Cells	Immune therapy response related factor	0.50	0.01	0.05
Naive B Cells	Immune therapy response related factor	0.49	0.01	0.05
PD-L1	Immune therapy response related factor	0.48	0.01	0.05
T cells follicular helper	Immune therapy response related factor	0.42	0.03	0.12
T cell inflamed GEP	Immune therapy response related factor	0.41	0.04	0.12
Tumor Mutational burden	irAE related factor/Immune therapy response related factor	0.40	0.04	0.13
Plasma cells	Immune therapy response related factor	0.39	0.05	0.13
SNV Neoantigen burden	Immune therapy response related factor	0.37	0.06	0.13
INDEL Neoantigen burden	Immune therapy response related factor	0.37	0.09	0.16
Dendritic cells resting	Immune therapy response related factor	0.34	0.09	0.16
IFN α response	irAE related factor/Immune therapy response related factor	0.33	0.10	0.17
Dendritic cells activated	Immune therapy response related factor	0.29	0.15	0.22
TNF signature	irAE related factor/Immune therapy response related factor	0.24	0.24	0.31
Intra tumor heterogeneity	Immune therapy response related factor	0.23	0.26	0.33
T cells regulatory Tregs	Immune therapy response related factor	0.16	0.43	0.51
Mast cells resting	Immune therapy response related factor	0.13	0.51	0.58
Neutrophils	irAE related factor/Immune therapy response related factor	0.13	0.54	0.59
NK cells activated	Immune therapy response related factor	0.04	0.85	0.88
CD4+ T Cells Memory Resting	Immune therapy response related factor	-0.01	0.97	0.97
Macrophages M0	Immune therapy response related factor	-0.07	0.72	0.77
Monocytes	Immune therapy response related factor	-0.22	0.28	0.35
CD4+ T cells naive	Immune therapy response related factor	-0.25	0.21	0.29
NK cells resting	Immune therapy response related factor	-0.30	0.14	0.21
Mast cells activated	Immune therapy response related factor	-0.32	0.11	0.18
B cells memory	Immune therapy response related factor	-0.32	0.11	0.18
PD-L1 protein expression	Immune therapy response related factor	-0.37	0.06	0.13
T cell Exhaustion Signature	Immune therapy response related factor	-0.38	0.05	0.13
Macrophages M2	Immune therapy response related factor	-0.39	0.05	0.13
T cells CD4+ memory activated	Immune therapy response related factor	NA	NA	NA
T cells γ δ	Immune therapy response related factor	NA	NA	NA
Eosinophils	irAE related factor/Immune therapy response related factor	NA	NA	NA

Supplementary Table 3. Combined effects of bivariate irAE predicting models for all combination of seven significantly correlated variables

Factor combination		Rs	P	FDR	log-likelihood ratio test (compare with factor 1)	log-likelihood ratio test (compare with factor 2)
Factor1	Factor2					
TCR diversity	CD8+ T Cells	0.75	1.7E-05	8.2E-04	0.02	0.02
PD-1	TCR diversity	0.68	2.2E-04	0.01	0.33	0.05
Cytolytic activity	TCR diversity	0.67	2.4E-04	0.01	0.42	0.02
IFN γ signature	CD8+ T Cells	0.67	2.6E-04	0.01	0.13	0.06
PD-1	Naive B Cells	0.67	2.8E-04	0.01	0.09	0.01
Cytolytic activity	CD8+ T Cells	0.66	3.0E-04	0.01	0.29	0.02
CD8+ T Cells	Naive B Cells	0.65	3.9E-04	0.01	0.01	0.00
Cytolytic activity	PD-1	0.65	4.4E-04	0.02	0.65	0.19
Cytolytic activity	IFN γ signature	0.65	4.9E-04	0.02	0.87	0.08
PD-1	Macrophages M1	0.63	7.5E-04	0.02	0.32	0.05
Cytolytic activity	Naive B Cells	0.63	7.6E-04	0.02	0.11	0.01
IFN γ signature	PD-1	0.62	8.5E-04	0.02	0.15	0.48
Cytolytic activity	Macrophages M1	0.61	1.14E-03	0.03	0.53	0.03
PD-1	CD8+ T Cells	0.61	1.25E-03	0.03	0.24	0.04
IFN γ signature	Macrophages M1	0.60	1.33E-03	0.03	0.71	0.22
Macrophages M1	CD8+ T Cells	0.60	1.57E-03	0.03	0.08	0.10
TCR diversity	Naive B Cells	0.58	2.20E-03	0.03	0.15	0.09
IFN γ signature	TCR diversity	0.57	2.72E-03	0.03	0.26	0.10
TCR diversity	Macrophages M1	0.54	4.59E-03	0.04	0.17	0.18
IFN γ signature	Naive B Cells	0.54	0.01	0.04	0.12	0.03
Macrophages M1	Naive B Cells	0.53	0.01	0.04	0.08	0.05

Supplementary Table 4. Combined effects of trivariate model for 7 significant variables

Bi-variate models	Add factor	Rs	P	log-likelihood ratio test vs bi-variate model
TCR diversity+CD8+ T Cells	Cytolytic activity	0.75	1.9E-05	0.27
TCR diversity+CD8+ T Cells	IFN γ signature	0.77	8.0E-06	0.60
TCR diversity+CD8+ T Cells	PD-1	0.72	5.2E-05	0.47
TCR diversity+CD8+ T Cells	Macrophage M1	0.73	3.5E-05	0.75
TCR diversity+CD8+ T Cells	Naïve B Cells	0.68	1.8E-04	0.08
CD8+ T Cells+Naïve B Cells	Cytolytic activity	0.73	3.9E-05	0.18
CD8+ T Cells+Naïve B Cells	PD-1	0.69	1.3E-04	0.30
CD8+ T Cells+Naïve B Cells	TCR diversity	0.68	1.8E-04	0.29
CD8+ T Cells+Naïve B Cells	IFN γ signature	0.67	2.9E-04	0.49
CD8+ T Cells+Naïve B Cells	Macrophage M1	0.64	6.0E-04	0.58

Supplementary Table 5. Multi-omics screen of irAE ROR significantly related factors

Category	Factor	Rs	P	FDR
Gene Expression	LCP1	0.82	3.5E-07	0.01
Gene Expression	GZMK	0.79	2.0E-06	0.02
Gene Expression	CRTAM	0.78	3.1E-06	0.02
Gene Expression	CD8A	0.77	4.0E-06	0.02
Gene Expression	ADPGK	0.76	6.7E-06	0.02
Gene Expression	SH2D1A	0.76	7.3E-06	0.02
Gene Expression	CTSW	0.76	8.2E-06	0.02
Gene Expression	HLA-DPB2	0.75	1.2E-05	0.02
Gene Expression	PTPRCAP	0.75	1.2E-05	0.02
Gene Expression	KIR3DL2	0.74	1.3E-05	0.02
Gene Expression	GPR18	0.74	1.7E-05	0.03
Gene Expression	CCL5	0.74	1.7E-05	0.03
Gene Expression	STAP1	0.73	2.0E-05	0.03
Gene Expression	KIAA0748	0.73	2.1E-05	0.03
Gene Expression	IL12B	0.73	2.3E-05	0.03
Gene Expression	TIGIT	0.73	2.6E-05	0.03
Gene Expression	PLA2G2D	0.72	2.9E-05	0.03
Gene Expression	TCL1A	0.72	2.9E-05	0.03
Gene Expression	LY9	0.72	3.5E-05	0.04
Gene Expression	CD96	0.72	3.9E-05	0.04
Gene Expression	CST7	0.72	4.0E-05	0.04
Gene Expression	CCR7	0.71	4.7E-05	0.04
Gene Expression	CD27	0.71	5.1E-05	0.04
Gene Expression	HLA-DQA2	0.71	5.6E-05	0.04
Gene Expression	SLFN5	0.71	5.8E-05	0.04
Gene Expression	FAIM3	0.70	6.5E-05	0.05
Gene Expression	SLC46A2	0.70	6.9E-05	0.05
Gene Expression	TRIM38	0.70	7.1E-05	0.05
Gene Expression	GPR174	0.70	7.5E-05	0.05
Gene Expression	BTLA	0.70	7.6E-05	0.05
Protein Expression	P70S6K_pT389	-0.67	2.6E-04	0.02
Protein Expression	CAVEOLIN1	0.66	3.1E-04	0.02
Protein Expression	P53	-0.66	3.6E-04	0.02
Protein Expression	TRANSGLUTAMINASE	0.62	1.0E-03	0.05

Supplementary Table 6. Combined effects of bivariate irAE predicting models for all combination of top 10 irAE ROR significantly correlated genes

Combination		Rs	P	FDR	log-likelihood ratio test vs factor1	log-likelihood ratio test vs factor2
Factor1	Factor2					
LCP1	ADPGK	0.91	7.9E-11	7.9E-09	0.05	2.8E-03
GZMK	ADPGK	0.89	8.4E-10	8.2E-08	0.02	2.7E-03
ADPGK	CTSW	0.88	2.3E-09	2.2E-07	0.01	0.02
ADPGK	SH2D1A	0.88	3.5E-09	3.3E-07	1.1E-03	0.01
ADPGK	KIR3DL2	0.86	1.3E-08	1.2E-06	2.5E-03	4.7E-03
LCP1	CTSW	0.86	1.5E-08	1.3E-06	0.02	1.8E-03
LCP1	CRTAM	0.86	2.0E-08	1.8E-06	0.02	0.01
LCP1	CD8A	0.85	3.3E-08	2.9E-06	0.03	0.01
CD8A	ADPGK	0.85	3.4E-08	2.9E-06	0.03	4.8E-03
CRTAM	ADPGK	0.85	4.2E-08	3.4E-06	0.02	2.7E-03
ADPGK	HLA.DPB2	0.85	5.1E-08	4.1E-06	0.01	0.02
LCP1	PTPRCAP	0.84	5.9E-08	4.6E-06	0.02	0.01
LCP1	GZMK	0.84	7.0E-08	5.3E-06	0.03	0.02
LCP1	SH2D1A	0.83	1.2E-07	8.9E-06	0.04	0.02
ADPGK	PTPRCAP	0.83	1.3E-07	9.1E-06	1.6E-03	0.02
LCP1	KIR3DL2	0.83	1.9E-07	1.3E-05	0.03	2.7E-03
LCP1	HLA.DPB2	0.82	2.3E-07	1.6E-05	0.03	3.7E-03
SH2D1A	HLA.DPB2	0.82	3.2E-07	2.1E-05	0.12	0.03
CRTAM	CTSW	0.81	6.3E-07	4.0E-05	0.55	0.06
GZMK	CTSW	0.81	6.7E-07	4.1E-05	0.43	0.05
CRTAM	CD8A	0.80	7.3E-07	4.3E-05	0.44	0.28
GZMK	CRTAM	0.80	8.1E-07	4.6E-05	0.36	0.34
GZMK	HLA.DPB2	0.80	8.5E-07	4.6E-05	0.24	0.05
HLA.DPB2	KIR3DL2	0.80	8.9E-07	4.7E-05	0.03	0.02
CRTAM	SH2D1A	0.80	9.8E-07	5.0E-05	0.26	0.29
SH2D1A	CTSW	0.80	1.1E-06	5.3E-05	0.36	0.04
CD8A	KIR3DL2	0.80	1.1E-06	5.3E-05	0.20	0.05
GZMK	KIR3DL2	0.79	1.3E-06	5.7E-05	0.31	0.05
CRTAM	KIR3DL2	0.79	1.3E-06	5.8E-05	0.16	0.03
CD8A	SH2D1A	0.79	1.6E-06	6.4E-05	0.32	0.59
CD8A	CTSW	0.79	1.6E-06	6.4E-05	0.56	0.08
CD8A	HLA.DPB2	0.79	1.6E-06	6.4E-05	0.19	0.06
SH2D1A	PTPRCAP	0.78	2.4E-06	8.2E-05	0.19	0.31
GZMK	CD8A	0.78	2.5E-06	8.2E-05	0.39	0.24
CTSW	HLA.DPB2	0.78	2.6E-06	8.2E-05	0.06	0.11
CTSW	KIR3DL2	0.78	2.9E-06	8.2E-05	0.05	0.07
GZMK	PTPRCAP	0.77	3.5E-06	8.7E-05	0.11	0.18
HLA.DPB2	PTPRCAP	0.77	3.5E-06	8.7E-05	0.03	0.18
CRTAM	PTPRCAP	0.77	3.5E-06	8.7E-05	0.15	0.26
PTPRCAP	KIR3DL2	0.77	4.0E-06	8.7E-05	0.19	0.02
GZMK	SH2D1A	0.77	4.2E-06	8.7E-05	0.31	0.33
CD8A	PTPRCAP	0.77	5.2E-06	8.7E-05	0.19	0.57
SH2D1A	KIR3DL2	0.76	6.1E-06	8.7E-05	0.23	0.04
CTSW	PTPRCAP	0.76	6.8E-06	8.7E-05	0.04	0.66
CRTAM	HLA.DPB2	0.76	8.2E-06	8.7E-05	0.46	0.09

Supplementary Table 7. Combined effects of trivariate model for top 10 significant genes.

Bivariate models	Add factor	Rs	P	log-likelihood ratio test vs bivariate model
LCP1+ADPGK	CTSW	0.912	8.7E-11	0.07
LCP1+ADPGK	GZMK	0.895	6.7E-10	0.07
LCP1+ADPGK	SH2D1A	0.895	7.0E-10	0.05
LCP1+ADPGK	KIR3DL2	0.889	1.3E-09	0.03
LCP1+ADPGK	CRTAM	0.888	1.4E-09	0.05
LCP1+ADPGK	CD8A	0.885	2.0E-09	0.08
LCP1+ADPGK	PTPRCAP	0.868	9.1E-09	0.03
LCP1+ADPGK	HLA.DPB2	0.858	2.2E-08	0.08

Supplementary Table 8. Combined effects of bivariate model for all combination of significant top 7 factors and top 10 genes

Factor combination		Rs	P	FDR	log likelihood ratio test (compare with factor 1)	log likelihood ratio test (compare with factor 2)
Factor1	Factor2					
CD8+ T Cells	LCP1	0.87	7.6E-09	2.8E-07	2.1E-04	0.12
Cytolytic activity	ADPGK	0.87	8.1E-09	2.8E-07	9.6E-03	0.03
TCR diversity	ADPGK	0.85	3.9E-08	9.1E-07	7.6E-04	0.03
PD-1	LCP1	0.84	9.8E-08	1.4E-06	8.3E-04	0.11
PD-1	ADPGK	0.84	1.1E-07	1.4E-06	6.9E-03	0.05
IFN γ signature	ADPGK	0.83	1.2E-07	1.4E-06	3.8E-03	0.07
Macrophages M1	LCP1	0.83	2.1E-07	2.1E-06	4.4E-04	0.39
IFN γ signature	LCP1	0.82	3.1E-07	2.8E-06	9.5E-04	0.40
Cytolytic activity	LCP1	0.81	4.5E-07	3.5E-06	1.3E-03	0.08
Macrophages M1	ADPGK	0.81	5.1E-07	3.6E-06	8.1E-04	0.03
CD8+ T Cells	GZMK	0.81	6.5E-07	4.1E-06	1.2E-03	0.53
CD8+ T Cells	HLA-DPB2	0.80	7.8E-07	4.2E-06	6.0E-04	0.04
TCR diversity	LCP1	0.80	7.8E-07	4.2E-06	3.6E-04	0.31
Macrophages M1	CRTAM	0.80	1.0E-06	5.1E-06	9.2E-04	0.41
Naive B Cells	ADPGK	0.80	1.1E-06	5.1E-06	5.6E-04	0.03
Naive B Cells	LCP1	0.80	1.2E-06	5.1E-06	2.5E-04	0.33
CD8+ T Cells	CRTAM	0.79	1.2E-06	5.1E-06	6.2E-04	0.19
PD-1	CD8A	0.79	1.7E-06	6.4E-06	4.8E-03	0.28
Macrophages M1	GZMK	0.79	1.7E-06	6.4E-06	1.1E-03	0.63
IFN γ signature	GZMK	0.79	2.0E-06	7.1E-06	2.7E-03	0.99
Naive B Cells	CD8A	0.78	2.2E-06	7.3E-06	6.3E-04	0.30
Cytolytic activity	CTSW	0.78	2.5E-06	7.6E-06	0.10	0.70
TCR diversity	CRTAM	0.78	2.5E-06	7.6E-06	1.2E-03	0.73
IFN γ signature	CRTAM	0.78	2.8E-06	7.7E-06	2.8E-03	0.92
PD-1	GZMK	0.78	2.8E-06	7.7E-06	6.4E-03	0.86
TCR diversity	CD8A	0.78	3.0E-06	7.8E-06	1.7E-03	0.88
IFN γ signature	CD8A	0.78	3.0E-06	7.8E-06	3.2E-03	0.57
Naive B Cells	HLA-DPB2	0.78	3.2E-06	7.9E-06	6.7E-04	0.10
Cytolytic activity	GZMK	0.78	3.3E-06	7.9E-06	0.01	0.54
Naive B Cells	CTSW	0.77	3.4E-06	7.9E-06	3.3E-04	0.03
IFN γ signature	CTSW	0.77	3.6E-06	8.1E-06	0.02	0.67
CD8+ T Cells	ADPGK	0.77	3.7E-06	8.1E-06	3.0E-03	0.10
Cytolytic activity	CD8A	0.77	4.0E-06	8.4E-06	0.02	0.53
Cytolytic activity	CRTAM	0.77	4.2E-06	8.7E-06	0.01	0.74
Naive B Cells	CRTAM	0.77	4.4E-06	8.9E-06	2.8E-04	0.15
Macrophages M1	CD8A	0.77	5.0E-06	9.8E-06	1.8E-03	0.91
IFN γ signature	KIR3DL2	0.76	5.5E-06	1.0E-05	8.7E-03	0.40
PD-1	CRTAM	0.76	6.0E-06	1.1E-05	6.5E-03	0.82
Naive B Cells	PTPRCAP	0.76	6.2E-06	1.1E-05	3.3E-04	0.33
TCR diversity	GZMK	0.76	6.2E-06	1.1E-05	1.1E-03	0.63
CD8+ T Cells	CD8A	0.76	6.9E-06	1.2E-05	2.0E-03	0.69
TCR diversity	CTSW	0.76	7.0E-06	1.2E-05	4.7E-03	0.36
Naive B Cells	GZMK	0.76	7.6E-06	1.2E-05	4.3E-04	0.28
Cytolytic activity	SH2D1A	0.76	7.9E-06	1.3E-05	8.3E-03	0.32

Factor combination		Rs	P	FDR	log likelihood ratio test (compare with factor 1)	log likelihood ratio test (compare with factor 2)
Factor1	Factor2					
TCR diversity	PTPRCAP	0.76	8.1E-06	1.3E-05	8.2E-04	0.89
CD8+ T Cells	KIR3DL2	0.75	8.5E-06	1.3E-05	6.6E-03	0.73
Macrophages M1	SH2D1A	0.75	8.9E-06	1.3E-05	1.1E-03	0.70
Macrophages M1	HLA-DPB2	0.75	9.2E-06	1.3E-05	5.0E-04	0.04
PD-1	PTPRCAP	0.75	9.2E-06	1.3E-05	1.5E-03	0.16
TCR diversity	SH2D1A	0.75	9.2E-06	1.3E-05	1.0E-03	0.63
CD8+ T Cells	SH2D1A	0.75	9.3E-06	1.3E-05	9.4E-04	0.37
TCR diversity	KIR3DL2	0.75	1.0E-05	1.4E-05	1.4E-03	0.11
TCR diversity	HLA-DPB2	0.75	1.1E-05	1.4E-05	1.7E-03	0.18
Cytolytic activity	PTPRCAP	0.75	1.1E-05	1.4E-05	9.5E-03	0.76
CD8+ T Cells	PTPRCAP	0.75	1.1E-05	1.5E-05	9.6E-04	0.74
Naive B Cells	SH2D1A	0.75	1.2E-05	1.5E-05	7.3E-04	0.75
PD-1	KIR3DL2	0.75	1.2E-05	1.5E-05	0.01	0.19
Cytolytic activity	HLA-DPB2	0.74	1.4E-05	1.6E-05	0.02	0.18
Macrophages M1	PTPRCAP	0.74	1.4E-05	1.6E-05	7.9E-04	0.67
PD-1	HLA-DPB2	0.74	1.4E-05	1.6E-05	9.9E-03	0.20
Macrophages M1	CTSW	0.74	1.6E-05	1.8E-05	4.1E-03	0.27
PD-1	CTSW	0.74	1.6E-05	1.8E-05	0.04	0.86
CD8+ T Cells	CTSW	0.74	1.9E-05	2.1E-05	7.1E-03	0.48
Cytolytic activity	KIR3DL2	0.73	2.4E-05	2.6E-05	0.05	0.44
PD-1	SH2D1A	0.73	2.4E-05	2.6E-05	4.6E-03	0.47
IFN γ signature	SH2D1A	0.73	2.4E-05	2.6E-05	1.5E-03	0.31
IFN γ signature	PTPRCAP	0.73	2.6E-05	2.7E-05	1.7E-03	0.72
IFN γ signature	HLA-DPB2	0.72	3.1E-05	3.2E-05	3.6E-03	0.17
Macrophages M1	KIR3DL2	0.72	3.4E-05	3.4E-05	4.9E-03	0.53
Naive B Cells	KIR3DL2	0.71	4.4E-05	4.4E-05	7.4E-04	0.08

Supplementary Table 9. Clinico-pathological character of patients

Patient ID	Cancer Type	Stages	irAEs	Drug	Line of treatment	Intervals*	LCP1	ADPGK	CD8	Geometric mean of LCP1 and ADPGK
1	lung cancer	IV	with irAEs	nivolumab	>=3	203	0.47	0.28	0.05	0.21
2	pancreas cancer	IV	with irAEs	nivolumab	>=3	82	0.45	0.23	0.04	0.09
3	lung cancer	IV	with irAEs	pembrolizumab	1	108	0.67	0.35	0.09	0.36
4	lung cancer	IV	with irAEs	nivolumab	2	13	0.69	0.27	0.15	0.32
5	lung cancer	IV	with irAEs	pembrolizumab	1	4	0.16	0.21	0.02	0.48
6	liver cancer	IV	with irAEs	pembrolizumab	2	20	0.20	0.14	0.23	0.09
7	lung cancer	IV	with irAEs	nivolumab	1	3	0.58	0.63	0.17	0.17
8	lung cancer	IV	with irAEs	durvalumab	1	7	0.44	0.46	0.02	0.44
9	lung cancer	III	with irAEs	nivolumab	1	10	0.73	0.53	0.38	0.41
10	lung cancer	IV	with irAEs	pembrolizumab	2	1	0.27	0.23	0.03	0.18
11	lung cancer	IV	with irAEs	pembrolizumab	2	11	0.87	0.44	0.02	0.03
12	lung cancer	IV	with irAEs	pembrolizumab	1	3	0.39	0.38	0.03	0.16
13	lung cancer	IV	with irAEs	pembrolizumab	>=3	58	0.14	0.08	0.19	0.60
14	lung cancer	IV	with irAEs	sintilimab	2	89	0.23	0.04	0.11	0.08
15	lung cancer	IV	without irAEs	nivolumab	>=3	69	0.42	0.10	0.02	0.45
16	lung cancer	IV	without irAEs	nivolumab	2	120	0.13	0.06	0.04	0.62
17	lung cancer	IV	without irAEs	pembrolizumab	2	94	0.45	0.37	0.05	0.25
18	lung cancer	III	without irAEs	atezolizumab	2	5	0.24	0.02	0.15	0.12
19	lung cancer	III	without irAEs	atezolizumab	2	5	0.22	0.06	0.16	0.62
20	lung cancer	III	without irAEs	pembrolizumab	2	29	0.26	0.15	0.05	0.38
21	lung cancer	IV	without irAEs	pembrolizumab	>=3	28	0.28	0.36	0.05	0.04
22	lung cancer	IV	without irAEs	nivolumab	>=3	23	0.35	0.16	0.05	0.07
23	lung cancer	IV	without irAEs	nivolumab	>=3	139	0.26	0.20	0.03	0.20
24	lung cancer	IV	without irAEs	pembrolizumab	2	119	0.40	0.16	0.06	0.32
25	lung cancer	IV	without irAEs	sintilimab	2	2	0.09	0.30	0.08	0.11
26	lung cancer	IV	without irAEs	nivolumab	1	161	0.03	0.05	0.00	0.24
27	lung cancer	IV	without irAEs	pembrolizumab	1	2	0.09	0.05	0.02	0.23
28	lung cancer	IV	without irAEs	nivolumab	1	36	0.10	0.01	0.00	0.26

*: The time period between tumor sample collection and anti-PD-1/PD-L1 therapy. Unit: weeks.
