# Supplementary material for: Multi-omics prediction of immune-related adverse events during checkpoint immunotherapy
Source: Nat Commun. 2020 Oct 2;11:4946. doi: 10.1038/s41467-020-18742-9 (PMC7532211; doi:10.1038/s41467-020-18742-9)
Supplement: Supplementary file 3 — Reporting Summary [file 41467_2020_18742_MOESM3_ESM.pdf]

nature research

Corresponding author(s): Leng Han

Last updated by author(s): Aug 23, 2020

Reporting Summary

Nature Research wishes to improve the reproducibility of the work that we publish. This form provides structure for consistency and transparency in reporting. For further information on Nature Research policies, see our Editorial Policies and the Editorial Policy Checklist.

Statistics

For all statistical analyses, confirm that the following items are present in the figure legend, table legend, main text, or Methods section.

n/a	Confirmed	
☐	☒	The exact sample size (*n*) for each experimental group/condition, given as a discrete number and unit of measurement
☒	☐	A statement on whether measurements were taken from distinct samples or whether the same sample was measured repeatedly
☐	☒	The statistical test(s) used AND whether they are one- or two-sided<br>*Only common tests should be described solely by name; describe more complex techniques in the Methods section.*
☐	☒	A description of all covariates tested
☐	☒	A description of any assumptions or corrections, such as tests of normality and adjustment for multiple comparisons
☐	☒	A full description of the statistical parameters including central tendency (e.g. means) or other basic estimates (e.g. regression coefficient) AND variation (e.g. standard deviation) or associated estimates of uncertainty (e.g. confidence intervals)
☐	☒	For null hypothesis testing, the test statistic (e.g. *F*, *t*, *r*) with confidence intervals, effect sizes, degrees of freedom and *P* value noted<br>*Give P values as exact values whenever suitable.*
☒	☐	For Bayesian analysis, information on the choice of priors and Markov chain Monte Carlo settings
☒	☐	For hierarchical and complex designs, identification of the appropriate level for tests and full reporting of outcomes
☒	☐	Estimates of effect sizes (e.g. Cohen's *d*, Pearson's *r*), indicating how they were calculated

Our web collection on statistics for biologists contains articles on many of the points above.

Software and code

Policy information about availability of computer code

Data collection	No software was used for data collection.
Data analysis	We performed leave-one-out cross-validation in predicting irAE ROR from bivariate and trivariate regression models using the R package caret v6.0. The goodness of fit of the models was compared by the log-likelihood ratio test using the R package lmtest v0.9. We used Variance inflation factor to assess multicollinearity by vif function of the car R package v3.0. Pathway enrichment was conducted using the R package clusterprofiler v 3.14. Calculation of the area under the ROC curve was completed by pROC R package v1.16. Anatomic illustration was generated by R package gganatogram v1.1. We used GSVA R package v1.3 to compute the T cell-inflammed gene expression profiling (GEP) level. Multiple comparisons were Benjamini-Hochberg adjusted by p.adjust function of the base R language, version 3.5.0. Spearman correlation testing was performed using the base R language, version 3.5.0. Whole slides were scanned with an Aperio ScanScope system (Leica Biosystems) and quantified using the Aperio ImageScope software v14.3 with Positive Pixel Count v9 (PPCv9) algorithm.

For manuscripts utilizing custom algorithms or software that are central to the research but not yet described in published literature, software must be made available to editors and reviewers. We strongly encourage code deposition in a community repository (e.g. GitHub). See the Nature Research guidelines for submitting code & software for further information.

Data

Policy information about availability of data

All manuscripts must include a data availability statement. This statement should provide the following information, where applicable:

- Accession codes, unique identifiers, or web links for publicly available datasets
- A list of figures that have associated raw data
- A description of any restrictions on data availability

All associated data are available in Source Data 1 and 2 for Figs. 1–3 and Supplementary Figs. 1–13, respectively. Individual safety records were downloaded from

FAERS database [https://www.fda.gov/drugs/questions-and-answers-fdas-adverse-event-reporting-system-faers/fda-adverse-event-reporting-system-faers-public-dashboard]. The TCGA data was downloaded from TCGA data portal [https://portal.gdc.cancer.gov/] and GDC PanImmune Data Portal [https://gdc.cancer.gov/about-data/publications/panimmune]. All the remaining data are available within the Article, Supplementary Information files or available from the author upon reasonable request.

Field-specific reporting

Please select the one below that is the best fit for your research. If you are not sure, read the appropriate sections before making your selection.

- [x] Life sciences
- [ ] Behavioural & social sciences
- [ ] Ecological, evolutionary & environmental sciences

For a reference copy of the document with all sections, see nature.com/documents/nr-reporting-summary-flat.pdf

Life sciences study design

All studies must disclose on these points even when the disclosure is negative.

Sample size

For the analysis of FAERS and TCGA data, we have described the sample size for each dataset in Supplementary Table 1. For the validation cohort, we included 28 patients and have described the cohort selection process and criteria in Online Methods section. We described sample size for all analyses clearly in the supplementary files.

Data exclusions

For retrospective patient cohort, we use three criteria to exclude patients in irAE group: 1) we only include patients with CT confirmed pneumonitis; 2) we only include the pneumonitis that require and respond to steroids, immunosuppressants or endocrine therapies; 3) two investigators independently determine the pneumonitis as immunologic etiology. We collected comparable number of anti-PD-1/PD-L1 treated cancer patients without any observed irAEs with matched cancer types, stage, age, sex, and therapy of irAE group. Patients with incomplete demographic and follow-up information, receiving anti-PD-1/PD-L1 in other hospitals, history of anti-CTLA-4 therapy, low-quality FFPE tumor samples, or tumor tissue contain >30% necrosis were excluded.

Replication

All data analyses are reproducible. All codes are carefully checked to ensure the replication.

Randomization

All the data is from public data resources or retrospective cohort. This is not relevant to our study.

Blinding

All the data is from public data resources or retrospective cohort. This is not relevant to our study.

Reporting for specific materials, systems and methods

We require information from authors about some types of materials, experimental systems and methods used in many studies. Here, indicate whether each material, system or method listed is relevant to your study. If you are not sure if a list item applies to your research, read the appropriate section before selecting a response.

Materials & experimental systems

n/a	Involved in the study	
[ ]	[x]	Antibodies
[x]	[ ]	Eukaryotic cell lines
[x]	[ ]	Palaeontology and archaeology
[x]	[ ]	Animals and other organisms
[ ]	[x]	Human research participants
[x]	[ ]	Clinical data
[x]	[ ]	Dual use research of concern

Methods

n/a	Involved in the study	
[x]	[ ]	ChIP-seq
[x]	[ ]	Flow cytometry
[x]	[ ]	MRI-based neuroimaging

Antibodies

Antibodies used

Cell signaling technology #3588 (D1C3) were used for LCP1 immunohistochemistry (1:200 diluted). Novus Biologicals #NBP1-91653 were used for ADPGK immunohistochemistry (1:900 diluted). MXB Biotechnology #RMA-0514 were used for CD8 immunohistochemistry (1:1 diluted). Horseradish peroxidase-conjugated secondary antibodies (1:200, NeoBioscience, #ANR02-1) were used.

Validation

The antibodies used in this study were validated for the species and applications by the indicated manufacturers.
LCP1: Cell Signaling Technology provides several references for validation. https://www.cellsignal.com/products/primary-antibodies/lcp1-d1c3-rabbit-mab/3588
ADPGK: Novus Biologicals provides several references for validation. https://www.novusbio.com/products/adpgk-antibody_nbp1-91653#datasheet
CD8: MXB Biotechnology provides several references for validation. http://www.maxim.com.cn/sitecn/dklkthdklkt/7013.html

Human research participants

Policy information about studies involving human research participants

Population characteristics	Our validation cohort consist of 28 cancer patients receiving anti-PD-1/PD-L1 inhibitors. The median age of the patients was 56 years (range, 37 to 82 years), with 22 (78.6%) male patients and 6 (21.4%) female patients. 26 of 28 (92.9%) patients were diagnosed as lung cancer.
Recruitment	Our study does not involve patient recruitment. Patients with high-quality formalin-fixed paraffin-embedded (FFPE) pre-treatment tumor tissues and clinicopathological information were retrospectively retrieved.
Ethics oversight	The study was conducted in accordance with ethical guidelines of U.S. Common Rule, and was approved by the Ethics Committee of Beijing shijitan hospital. Written informed consent was obtained from all patients.

Note that full information on the approval of the study protocol must also be provided in the manuscript.
